# Proposal of a modified American Joint Committee on Cancer staging scheme for resectable pancreatic ductal adenocarcinoma with a lymph node ratio-based N classification: A retrospective cohort study

**DOI:** 10.1097/MD.0000000000012094

**Published:** 2018-08-24

**Authors:** Huan-Jun Li, Yu-Tong Chen, Shu-Qiang Yuan

**Affiliations:** a Department of Medical Oncology, Dongguan General Hospital, Dongguan; b Department of Medical Oncology, Sun Yat-Sen University Cancer Center; c State Key Laboratory of Oncology in South China; d Collaborative Innovation Center for Cancer Medicine; e Department of Gastric and Pancreatic Surgery, Sun Yat-Sen University Cancer Center, Guangzhou, China.

**Keywords:** American Joint Committee on Cancer staging, lymph node ratio, pancreatic ductal adenocarcinoma, Surveillance, Epidemiology, and End Results

## Abstract

The recently launched 8th edition of the American Joint Committee on Cancer (AJCC) staging scheme for pancreatic ductal adenocarcinoma (PDAC) did not account for the impact of the total examined lymph node count on prognostic accuracy. In this population-based cohort study, we proposed a modified AJCC staging scheme by incorporating a lymph node ratio (LNR)-based N classification for patients with resectable PDAC.

We analyzed 8615 patients with resectable PDAC from the Surveillance, Epidemiology, and End Results database between 2004 and 2013. The optimal cut-off points for LNR were identified by recursive partitioning, and an LNR-based N classification was designed accordingly.

The LNR-based N classification could further stratify patients with the 8th AJCC N1 and N2 disease into subgroups with significantly different overall survival (*P* < .001 for both). By replacing the 8th AJCC N classification with the corresponding LNR-based N classification, we further proposed a modified AJCC staging scheme. The modified AJCC staging outperformed the 8th AJCC staging in terms of the discriminatory capacity measured by the concordance index and Akaike information criterion, and the prognostic homogeneity assessed by using the likelihood ratio chi-squared test and stratified survival analysis.

Replacing the 8th AJCC N classification with the LNR-based N classification can improve the prognostic performance of the 8th AJCC staging scheme for PDAC.

## 1 Introduction

Pancreatic ductal adenocarcinoma (PDAC) is the most common malignancy in the pancreas and the 7th-leading cause of cancer-related death around the world.^[1]^ With an average life expectancy on diagnosis of 4 to 6 months, PDAC is considered to be the worst of all gastrointestinal malignancies.^[2–4]^ Despite that radical resection offers the only chance for cure to patients with resectable PDAC, these patients have high incidence of postsurgical recurrence and dismal prognosis.^[5]^

In clinical practice, the prognosis of resectable PDAC is predicted based on the American Joint Committee on Cancer (AJCC) tumor-node-metastasis (TNM) classification, which involves the tumor invasion depth, lymph node status, and distal metastasis.^[6]^ However, the N classification of the AJCC staging scheme for PDAC is questioned by several studies.^[7–9]^ In the 7th edition of the AJCC staging scheme, patients with nodal metastasis are assigned to a single prognostic group—the N1 classification—regardless of the positive lymph node (PLN) count.^[6]^ In addition, the total examined lymph node (ELN) count is considered to be of great importance to staging accuracy for pancreatic cancer; that is, an increased number of ELNs is reported to decrease the possibility of stage migration and improves the oncological outcome.^[10]^ Although the recently launched 8th edition of the AJCC staging scheme for PDAC further divided the N1 classification of the 7th edition into N1 (1–3 PLNs) and N2 (≥4 PLNs), it still did not account for the impact of the ELN on prognostic accuracy.^[11]^

The lymph node ratio (LNR, i.e., the ratio of the number of PLNs to the number of total ELNs) is an emerging factor associated with prognosis in a variety of cancers including PDAC.^[12–16]^ It combines the prognostic impact of both the PLN and ELN counts and serves as a promising predictor of survival. In several previous studies of patients with PDAC, LNR showed a superior prognostic performance to PLN.^[17–19]^ However, to the best of our knowledge, none of these studies has incorporated the LNR system in the AJCC TNM staging for comprehensive evaluation.

In the present study of patients with resectable PDAC from the Surveillance, Epidemiology, and End Results (SEER) database, we developed a modified AJCC staging scheme by replacing the 8th AJCC N classification with an LNR-based N classification. We then comprehensively assessed whether the performance of this modified AJCC staging scheme was superior to the 8th AJCC staging scheme in terms of the discriminatory ability and prognostic homogeneity.

## 2 Patients and methods

### 2.1 Study cohort

From the National Cancer Institute's SEER database (18 SEER registries), we identified 72,700 aged 18 and older patients with PDAC (North American Association of Central Cancer Registries [NAACCR] item no. 400, codes: C25.0–C25.9 and NAACCR item no. 522, codes: 8140, 8150, 8210, 8211, 8251, 8260, 8261, 8263, 8480, 8481, 8490, 8500, and 8503) from January 2004 to December 2013. Patients without histologic diagnosis, without resection of the primary tumor, with a history of prior or concurrent malignancies, with carcinoma in situ, locally unresectable tumor (T4 classification), or distant metastasis, and with missing information regarding tumor size, the PLN count, or the ELN count were excluded. The final analytic cohort consisted of 8615 patients. All patients were restaged on the basis of the 8th AJCC staging scheme. Because SEER is public-use data, this study was deemed exempt from institutional review board approval by the Dongguan General Hospital and Sun Yat-Sen University Cancer Center, and informed consent was waived.

### 2.2 Statistical analysis

Overall survival (OS), measured from the time of initial diagnosis to the time of death from any cause or the time of censor, was the primary outcome of interest. The Kaplan–Meier method and log-rank test were used to compare OS among different groups. Multivariable Cox regression was used to examine the association between staging schemes and hazard ratios (HRs) for death after adjusted for baseline covariates including race, year of diagnosis, age, sex, marital status, SEER region, tumor site, and tumor grade.

Recursive partitioning was performed to determine the optimal cut-off points for LNR. This technique can objectively divide patients into different categories on the basis of LNR, which provides maximum survival discrimination and yields subgroups with relatively homogeneous survival performance.^[20,21]^ On the basis of recursive partitioning, 2 cut-off points were identified for LNR: 0.05 and 0.45. Accordingly, we defined the LNR-based N (rN) classification with the following 3 groups: rN0 (LNR ≤ 0.05), rN1 (0.05 < LNR ≤ 0.45), and rN2 (LNR > 0.45). By replacing the 8th AJCC N classification with the corresponding rN classification, we proposed a modified LNR-based AJCC (rAJCC) staging scheme with rIA, rIB, rIIA, rIIB, and rIII stages. The rAJCC staging scheme was internally validated using bootstraps with 1000 resamples to measure model overfit.

The performance of the rAJCC staging scheme was compared to the 8th AJCC staging scheme in terms of discriminatory ability and prognostic homogeneity. The discriminatory abilities of the 2 staging schemes were quantified using the concordance index (C-index)^[22]^ and Akaike information criterion (AIC). The higher the C-index or the lower the AIC value, the greater was the discriminatory capacity of the staging scheme. Likelihood ratio chi-squared tests related to the Cox regression model were used to measure the prognostic homogeneity of the staging schemes. The greater the likelihood ratio chi-squared value, the better prognostic homogeneity of the staging scheme. Stratified survival analyses, which investigated the prognostic effect of a staging scheme within the substages of the other staging scheme, were also performed to evaluate the prognostic homogeneity of the staging schemes.

Statistical significance was set at *P* < .050 in 2-tailed tests. All statistical analyses were performed using IBM SPSS Statistics for Windows v. 20.0 (IBM Corp., Armonk, NY) and R v. 3.3.1 (http://www.r-project.org).

## 3 Results

### 3.1 Clinicopathologic characteristics of the study cohort

Table 1 summarizes the characteristics of the study cohort (8615 cases). Most tumors (77.5%) originated from the head of pancreas. Additionally, most patients were diagnosed with T2 disease (58.2%) and N1 disease (42.0%) on the basis of the 8th AJCC staging scheme. The median PLN and ELN counts were 1 (interquartile range [IQR], 0–3) and 14 (IQR, 9–20), respectively. The median value of LNR was 0.1 (IQR, 0–0.25). The mean follow-up duration for survivors was 34.7 ± 25.6 months. The median OS for the study cohort was 19 months (95% confidence interval [CI], 18.5–19.5).

**Table 1:**
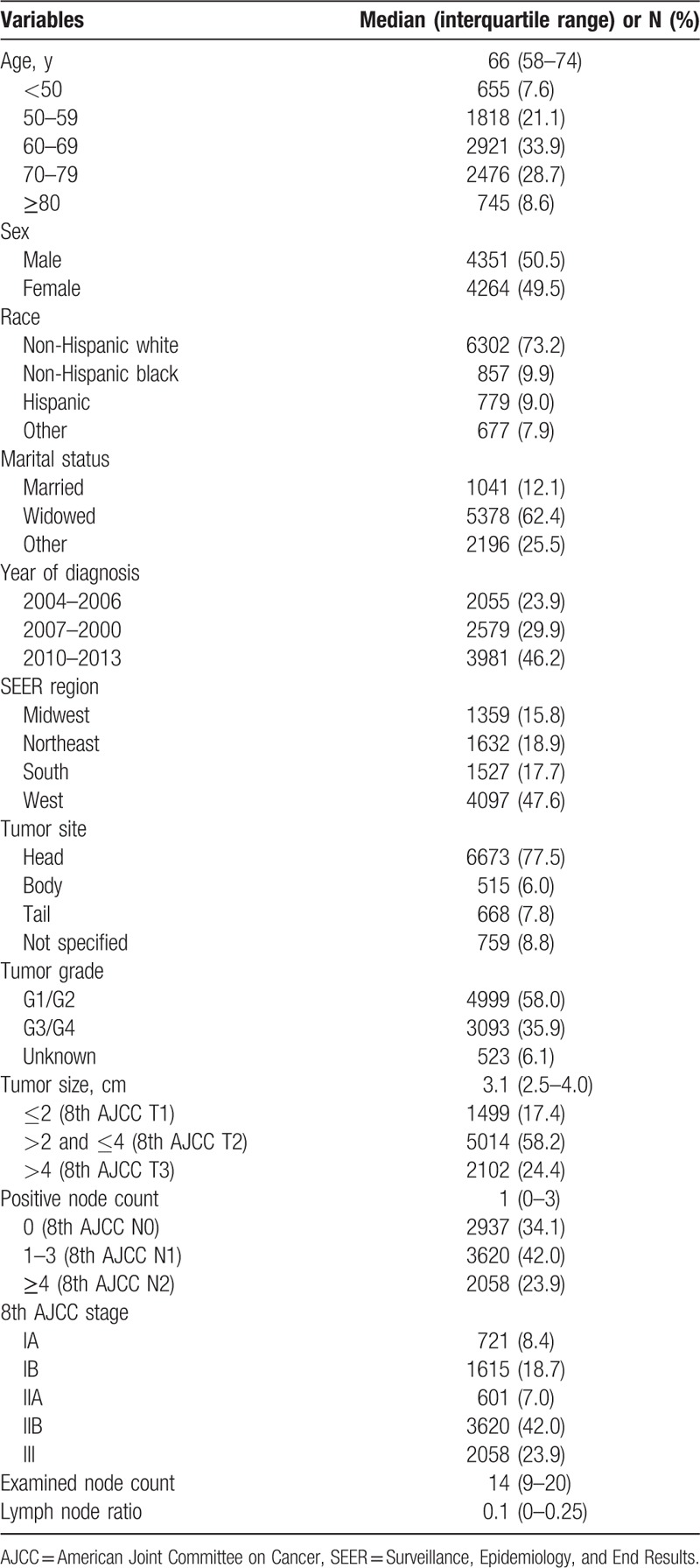
Clinicopathologic features of the study population.

### 3.2 Comparison of the prognostic performance of the 8th AJCC N classification and the rN classification

**Figure 1:**
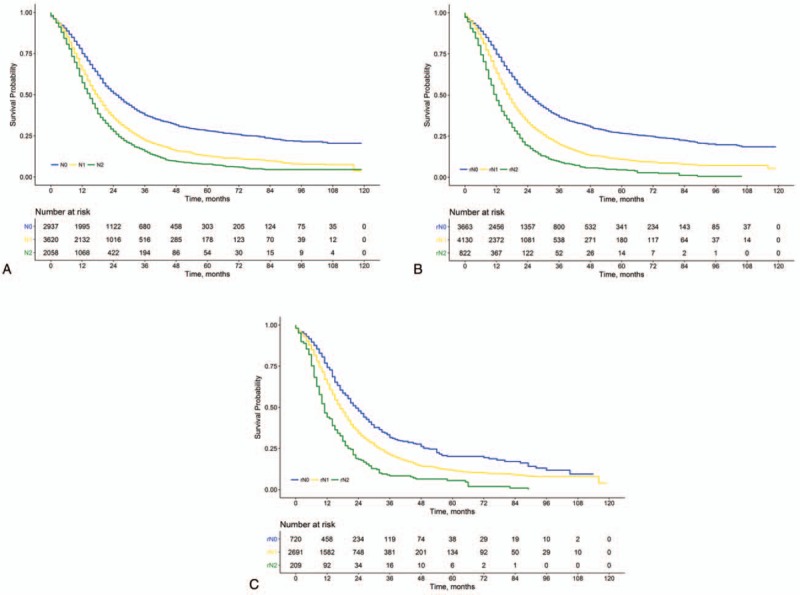
Overall survival for patients with resectable PDAC stratified by the 8th AJCC N classification (A) and the rN classification (B), and for patients with 8th AJCC N1 disease stratified by the rN classification (C). AJCC = American Joint Committee on Cancer, PDAC = pancreatic ductal adenocarcinoma.

Survival as determined using the N classification of the 8th AJCC staging scheme is presented in Fig. 1A. The median OS differed significantly among the three 8th AJCC N classifications (25, 18, and 15 months, respectively, for N0, N1, and N2; *P* < .001 for all pair-wise comparisons). The rN0, rN1, and rN2 stage groups included 3663 (42.5%), 4130 (47.9%), and 822 (9.5%) patients, respectively. The corresponding median OS for these groups was 25, 17, and 12 months, respectively (*P* < .001; Fig. 1B). Each rN stage group represented a distinct prognosis (*P* < .001 for all pair-wise comparisons).

The rN classification achieved a C-index of 0.578 (95% CI, 0.568–0.584), which was significantly superior to the 8th AJCC N classification (0.566 [95% CI, 0.558–0.574]; *P* = .012). Moreover, the rN classification outperformed the 8th AJCC N classification in terms of the AIC (95,409.0 vs 95,525.0) and in the likelihood ratio chi-squared test (likelihood ratio chi-squared value, 501.2 vs 385.3).

We further performed stratified survival analyses to investigate the prognostic effect of the rN classification within each 8th AJCC N classification and vice versa. Using the rN classification, patients with 8th AJCC N1 and N2 disease could be further stratified into subgroups with significantly different median OS (Table 2). Notably, patients with 8th AJCC N1 disease could be further stratified into the rN0, rN1, and rN2 subgroups, and a difference of 12 months in median OS was found between patients with rN0 and those with rN1 disease (23 vs 11 months, *P* < .001; Fig. 1C and Table 2). By contrast, all rN classifications exhibited favorable prognostic homogeneity when stratified by the 8th AJCC N classification (Table 2).

**Table 2:**
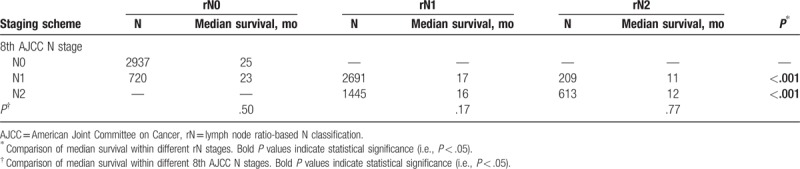
Comparison of prognostic homogeneity between the 8th AJCC N staging and the rN staging schemes.

### 3.3 Comparison of prognostic performance between the 8th AJCC staging and the rAJCC staging

**Figure 2:**
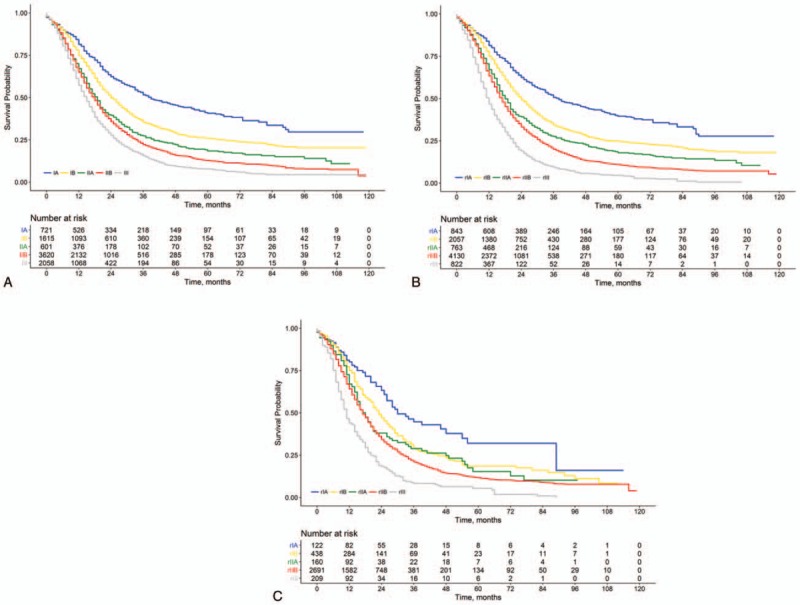
Overall survival for patients with resectable PDAC stratified by the 8th AJCC staging scheme (A) and the rAJCC staging scheme (B), and for patients with 8th AJCC IIB disease stratified by the rAJCC staging scheme (C). AJCC = American Joint Committee on Cancer, PDAC = pancreatic ductal adenocarcinoma, rAJCC = lymph node ratio-based AJCC.

Survival on the basis of the 8th AJCC stages is presented in Fig. 2A. The median OS differed significantly among the 5 stages (39, 24, 19, 18, and 15 months, respectively, for stages IA, IB, IIA, IIB, and III; *P* < .05 for all pair-wise comparisons). The rIA, rIB, rIIA, rIIB, and rIII stage groups of the modified AJCC staging included 843 (9.8%), 2057 (23.9%), 763 (8.9%), 4130 (47.9%), and 822 (9.5%) patients, respectively. The corresponding median OS for the rAJCC stages were 38, 24, 18, 17, and 12 months, respectively (*P* < .05 for all pair-wise comparisons; Fig. 2B). Multivariable Cox regression analysis confirmed that a higher rAJCC stage was associated with an increased risk of death (rIB vs rIA: HR, 1.45 [95% CI, 1.29–1.63]; rIIA vs rIA: HR, 1.84 [95% CI, 1.61–2.10]; rIIB vs rIA: HR, 2.21 [95% CI, 1.98–2.45]; rIII vs rIA: HR, 3.37 [95% CI, 2.97–3.82]; *P* < .001 for all).

The rAJCC staging scheme had a C-index of 0.585 (95% CI, 0.577–0.593). In the internal validation using bootstrapped resampling, the corrected C-index was also 0.585, indicating minimal evidence of model overfit. The rAJCC staging showed significantly greater discriminatory power than the 8th AJCC staging system (C-index, 0.585 [95% CI, 0.577–0.593] vs 0.573 [95% CI, 0.564–0.581]; *P* = .002). It also outperformed the 8th AJCC staging in terms of the AIC (95,316.2 vs 95,445.5) and in the likelihood ratio chi-squared test (likelihood ratio chi-squared value, 598.0 vs 468.9).

We then examined the prognostic effect of the rAJCC staging within each 8th AJCC stage. Patients with 8th AJCC IIB and III disease could be further stratified into subgroups with remarkably different median OS (Table 3). Of note, patients with 8th AJCC IIB disease could be further classified into the rIA, rIB, rIIA, rIIB, and rIII subgroups, and there was a difference of 24 months in median OS between the patients classified as having rIA disease and those classified as having rIII disease (35 vs 11 months, *P* < .001; Fig. 2C). Moreover, we further examined the homogeneity in survival within each rAJCC stage when assessed against the 8th AJCC staging. For patients within each rAJCC stage, survival was found to be homogeneous when stratified by 8th AJCC stages (Table 3). Of note, a fraction of the patients who were classified into 8th AJCC stages IB and IIB (median OS, 24 and 18 months, respectively; *P* < .001), actually had similar survival (median OS, 24 and 23 months, respectively; *P* = .17) and were both classified into stage rIB (Table 3).

**Table 3:**
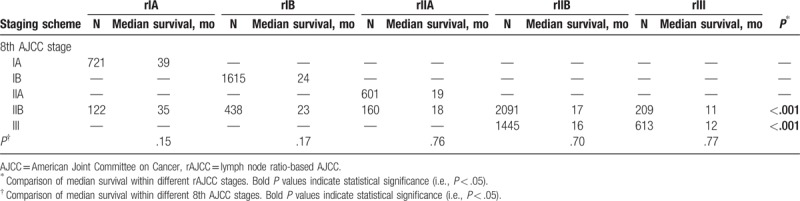
Comparison of prognostic homogeneity between the 8th AJCC staging and the rAJCC staging schemes.

## 4 Discussion

The primary flaw of the 8th AJCC N classification for PDAC is that its prognostic accuracy is significantly influenced by total ELN. A higher ELN count was reportedly associated with a higher PLN count and a decreased risk of false downstaging of the N classification.^[10]^ Previous evidence has suggested that LNR, which accounts for both the PLN and ELN counts, exhibits prognostic superiority to PLN.^[17–19]^ In this population-based study, we demonstrated significant prognostic heterogeneity within the 8th AJCC N1 and N2 disease after stratified by the rN classification, which verifies the prognostic value of LNR and suggests a room for improvement in the 8th AJCC staging scheme. Subsequently, we derived a novel rAJCC staging scheme for resectable PDAC by replacing the 8th AJCC N classification with the corresponding rN classification.

The rAJCC staging scheme outperformed the 8th AJCC staging scheme in terms of discriminatory power and prognostic homogeneity. Although the discrimination was only moderately better, the prognostic homogeneity was considerably better for the rAJCC staging scheme. On the one hand, OS was homogeneous within each rAJCC stage regardless of the 8th AJCC stages. For example, a fraction of the patients who were considered having different survival outcomes and classified into 8th AJCC stages IB or IIB, actually had similar survival (median OS, 24 and 23 months, respectively) and were both classified into stage rIB. On the other hand, stages IIB and III according to the 8th AJCC staging scheme could be classified by the rAJCC system into subgroups with remarkably different OS rates. For instance, patients with 8th AJCC IIB disease could be further classified into the rIA, rIB, rIIA, rIIB, and rIII subgroups, and a difference in median OS exceeding 20 months was detected between the patients classified as having rIA disease and those classified as having rIII disease (35 vs 11 months).

The proposed rAJCC staging scheme is of great clinical importance under the current treatment modality of resectable PDAC. Curative surgery followed by adjuvant chemotherapy is recommended by the National Comprehensive Cancer Network guidelines for resectable PDAC. However, the optimal chemotherapy regimen remains inconclusive and the OS benefit from adjuvant chemotherapy was reportedly modest in previous phase 3 trials.^[23–26]^ The proposed rAJCC staging scheme, which exhibited substantial prognostic superiority to the 8th AJCC staging, will be clinically useful in treatment planning, such as the decision making regarding the use of adjuvant chemotherapy as well as chemotherapy regimens. Furthermore, it may also help improve risk stratification of patients entering future clinical trials.

Several prognostic nomograms combining the prognostic information of various prognostic factors have been proposed to improve the prognostic accuracy for PDAC patients.^[27,28]^ Nevertheless, these nomograms have not been widely acknowledged, probably because they are inherently complex and inconvenient to use. On the contrary, the proposed rAJCC staging scheme is as simple as the 8th AJCC TNM classification. Thus, the rAJCC staging scheme is likely to be clinically practical considering its improved prognostic performance over the 8th AJCC staging scheme was not at the cost of complexity and that it was easy for use in outcome prediction and treatment planning.

Several limitations should be acknowledged with our study. Miscoding of data in the SEER database could still exist despite the great efforts being made to ensure the accuracy and quality of data. In addition, the SEER database does not cover the information on patient comorbidities and performance status, extent of lymphadenectomy and chemotherapy. Because OS is the primary endpoint in this study, medical comorbidities or other competing causes of death might influence our results. Still, OS is the most valuable endpoint for cancer patients and has a consistent definition across different institutions. Moreover, as information regarding chemotherapy was not available in the SEER database, future studies are needed to investigate how the proposed rAJCC staging may influence decision making regarding postoperative therapies. Last but not least, external validation using prospective cohorts as well as patient cohorts from other countries outside North America is required.

In conclusion, by replacing the 8th AJCC N classification with the rN classification, the proposed rAJCC staging scheme exhibited remarkable prognostic superiority to the 8th AJCC staging scheme for patients with resectable PDAC, without substantial increase in complexity. The rAJCC staging system may serve as a clinically useful tool for prognosis, surveillance, and treatment planning as well as risk stratification in future clinical trials for patients with PDAC.

## Acknowledgments

The authors thank the staff members of the National Cancer Institute and their colleagues across the United States and at Information Management Services, Inc., who have been involved with the SEER Program.

## Author contributions

**Conceptualization:** Huan-Jun Li, Yu-Tong Chen, Shu-Qiang Yuan.

**Data curation:** Huan-Jun Li, Yu-Tong Chen, Shu-Qiang Yuan.

**Formal analysis:** Huan-Jun Li, Yu-Tong Chen, Shu-Qiang Yuan.

**Investigation:** Huan-Jun Li, Yu-Tong Chen, Shu-Qiang Yuan.

**Software:** Huan-Jun Li, Yu-Tong Chen, Shu-Qiang Yuan.

**Visualization:** Huan-Jun Li.

**Writing – original draft:** Huan-Jun Li, Yu-Tong Chen, Shu-Qiang Yuan.

**Writing – review & editing:** Huan-Jun Li, Yu-Tong Chen, Shu-Qiang Yuan.
